# The extracellular polysaccharide inhibit porcine epidemic diarrhea virus with extract and gene editing *Lacticaseibacillus*

**DOI:** 10.1186/s12934-023-02226-8

**Published:** 2023-11-04

**Authors:** Shaojun Chen, Zida Nai, Ziliang Qin, Gang Li, Xinmiao He, Wentao Wang, Yaguang Tian, Di Liu, Xinpeng Jiang

**Affiliations:** 1 Northeast Agricultural University, Harbin, 150030 Heilongjiang People’s Republic of China; 2 Key Laboratory of Combining Farming and Animal Husbandry, Ministry of Agriculture, Animal Husbandry Research Institute, Heilongjiang Academy of Agricultural Sciences No, 368 Xuefu Road, Harbin, 150086 People’s Republic of China; 3 Undergraduate Experimental and Teaching Center, College of Animal Science and Technology, Northeast Agricultural University, Harbin, 150030 Heilongjiang People’s Republic of China; 4 Yanbian University, Yanji, 133002 Jilin People’s Republic of China

**Keywords:** The extracellular polysaccharide, The anti-PEDV infection, IFN-λ, Extract, Genome editing *lacticaseibacillus casei*

## Abstract

*Lacticaseibacillus* is one of the predominant microorganisms in gut from human and animal, and the *lacticaseibacillus* have effective applications against the viral diarrhea of piglets in the farm. However, the function and the concrete cell single pathways of the active ingredient from *lacticaseibacillus* was not clear within anti-infection in the postbiotics research. Here, we compared the biological function of extracellular polysaccharides (EPS) purified from *lacticaseibacillus casei* (*L. casei*) and gene editing *lacticaseibacillus casei* with the CRISPER-Cas9 technology, which were with the ability of antioxidation and anti-inflammation, and the EPS could also inhibit the ROS production within the Porcine Small Intestinal Epithelial Cells-J2 (IPEC-J2). Interestingly, we found that both of EPS and genome editing *lacticaseibacillus casei* could specifically target the IFN-λ expression in the IPEC-J2, which was beneficial against the PEDV infection in the virus replication and production with the qRT-PCR and indirect immunofluorescence methods. Finally, the STAT3 cell single pathway was stimulated to transcribe IFN-λ with the EPS to elucidate the detailed mechanism of activating type III IFN signals receptor of IL-10R2, which play the function between anti-inflammation and anti-virus in the PEDV infection. Taken together, our research linked a postbiotics of EPS with the antiviral infection of PEDV, which suggest that the *lacticaseibacillus* itself still have displayed the potential immunomodulatory activities, and highlight the immunomodulatory potential of EPS-producing microbes.

**Graphical Abstract:**

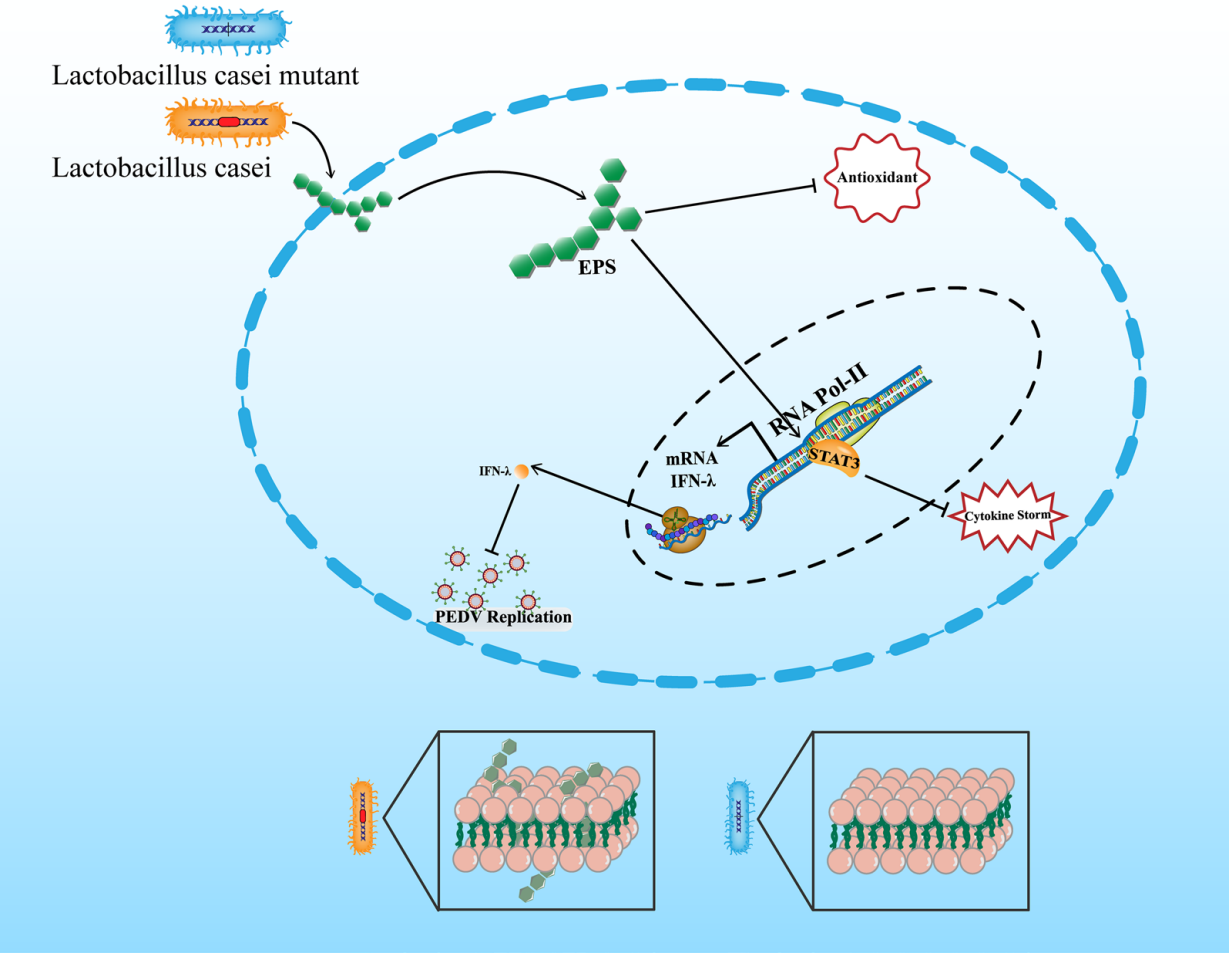

## Introduction

Probiotics, *lacticaseibacillus and bifidobacteria*, are main colonization in intestinal tract playing a beneficial effect on the human and animals. *Lacticaseibacillus* was a useful supplement for clinical therapeutics during acute diarrhea and intestinal inflammation [26]. A great number of researches focused on the function of *lacticaseibacillus* against intestinal infection, and the concrete cell single pathways have been drawn with the whole *lacticaseibacillus*, such as IL-10, NF-κB and Th1\Th2 [17–19, 21]. In the postbiotics era, the substance is released by or produced through the metabolic activity of the microorganism, such as short-chain fatty acids, surface proteins, bacteriocins, and polysaccharides, which exerted a beneficial effect on the host, directly or indirectly. The researchers aimed to find the active ingredient of *lacticaseibacillus*, which was endowed with unique function and concreting cell single mechanism in the gut disease from host [34, 41]. However, the purified the ingredient may not accurately defined the function comparing with the probiotic stimulating the intestinal mucosal immune system in the complexed microorganism environment of gut. The development of the genome editing technology in the *lacticaseibacillus* provide the possibility [6, 40], which further demonstrated the functionality of metabolic activity for the specific gene, and built the correlation comparing with deletional *lacticaseibacillus* and wild *lacticaseibacillus* with the specific gene in the gut of host. The live gene-edited *lacticaseibacillus* would much better to study the colonization, differentiation and metabolism comparing with the wild *lacticaseibacillus* in the gut. Among all ingredients, the *lacticaseibacillus* polysaccharide derived immunomodulatory effects, such as *lacticaseibacillus. casei* LOCK 0919 [12], *lacticaseibacillus paracasei* DG [3] and *lacticaseibacillus reuteri* ASM20016 [39, 42], which mainly comes to host response modulation through extracellular polysaccharides(EPS) [27]. However, all these researches were focused on the purified EPS in the vitro and cell experiment, there was not gene knock-out of *lacticaseibacillus* to explain all the function of substance released by or produced through the metabolic activity in host.

Most of the coronavirus were mainly susceptible to the epithelial cell, such as respiratory epithelial cells and intestinal epithelial cells, which was immune evasion with types I and II IFN. However, the type III IFN-lambda (IFN-λ) has been specific expression on the mucosal surfaces, including epithelial surfaces of the liver, respiratory, and gastrointestinal epithelial cell, and plays vital roles in controlling coronavirus infection within mucosal surfaces, such as SARS-CoV-2, COVID-19, MERS-CoV, SADS-CoV and PEDV from human and pigs [5, 10, 16, 44, 51].Thus, nutritional recommendations to prevent the COVID-19 infection with the oral administration of prebiotics and probiotics, since an imbalance in the intestinal microbiota with other viral infectious diseases [47], the prebiotics intervention reduce the risk of secondary infection and inflammatory storm with the viral infection. The extracellular extracts and cell wall fractions of *lactic acid bacteria* could provide protective effects against PEDV infections [7]. *Lacticaseibacillus* plantarum metabolites, the main component was exopolysaccharides, which prevented PEDV adsorption, and alleviated inflammatory responses and early apoptosis in the injured cells, but it could not regulate the immune function in the IPEC-J2 [14]. However, it remained unclear about viral clearance patterns with the oral administration of prebiotics after the coronavirus infection within the complexed microorganism environment of the host. Therefore, the active ingredient of *lacticaseibacillus* was required to design a following up research in the anti-viral infection. Most importantly, the mechanism of the exopolysaccharides from the *lacticaseibacillus* against the PEDV infection in the host of piglets.

Our previous study has found that the *lacticaseibacillus casei* could effectively inhibit diarrhea and inflammation with PEDV infection. And we have purified the EPS from the fermentation of *lacticaseibacillus casei*. The gene-edited *lacticaseibacillus casei*, which knock-out the glucose-1-phosphate thymidylyltransferase gene resulting in the decrease in exopolysaccharide (EPS) production, both of EPS and gene-edited *lacticaseibacillus casei* were used to study the molecular function to inhibit the PEDV infection with the IPEC-J2. And the in vitro experiments in the IPEC-J2 were used to evaluate the active ingredient of EPS, and which was also to assess the cell single pathway such as anti-inflammation and inhibition of viral replicationbetween polysaccharides and host cells with their potential immunomodulatory activities.

## Materials and methods

### The extraction of bacterial polysaccharides and gene-edited *Lacticaseibacillus casei*

*Lacticaseibacillus casei* (supplied by Brightdairy Dairy Co., Ltd, Shanghai, China) was grown in Man, Rogosa, Sharpe (MRS) broth for 1 L at 37 °C for 18 h without shaking. The supernatant was collected, and the polysaccharide antigens were precipitated with 5 volumes of cold 96% ethanol in 4 °C for overnight. The precipitate was suspended in water dialyzing for 48 h in water, and then freeze-drying. The precipitate of *lacticaseibacillus casei* was washed by PBS for two times. The EPS were performed by acidic phenol (Sigma-Aldrich) at 68 °C. The DNase I, Rnase and Pronase were used to remove nucleic acids and proteins at 37 °C, respectively. The pre-cooled ethanol was added to the polysaccharides, and then dialyzed against distilled water for 3 days and freeze-dried. Purified polysaccharides were dissolved in water for the next experiments.

The CRISPR-CAS9 gene editing plasmid pLCNICK were purchased from Addgene Company (ID: 84653), which was studied to decrease the production of polysaccharides in the *lacticaseibacillus casei* [40]. The pLCNICK plasmid was transferred into the *lacticaseibacillus casei* with the electroporation technology. To confirm the glucose-1-phosphate thymidylyltransferase gene deletion in the genome, we extracted the genome of gene edited *lacticaseibacillus casei*. Meanwhile, we amplified the Has of glucose-1-phosphate thymidylyltransferase gene of genome with PCR method, which demonstrated the sequence determination, glucose-1-phosphate thymidylyltransferase gene producing the EPS has been deleted in the genome. The genome editing *lacticaseibacillus casei* was used in our further experiments.

### Cytotoxic experiments

The IPEC-J2 was used to analyze the cytotoxicity, the best concentration of polysaccharide was chosen for the next experiments. Firstly, the extracellular polysaccharides (EPS) was isolated from cultured *lacticaseibacillus casei*. Then we added EPS in the IPEC-J2 culture media which the concentration of polysaccharides increased gradually from 25 μg/mL to 400 μg/mL. Then we measured the activity of IPEC-J2 for the maximum dose of cell cytotoxicity test with the CKK8 method.

### Cell antioxidation and oxidative damage assay

The experiment utilized a comparison control group designed with three groups of EPS(EPS + IPEC-J2) group, the *lacticaseibacillus* strain(*Lacticaseibacillus* + IPEC-J2) group and controls group with IPEC-J2 group. Briefly, the supernatants were collected and stored at − 80 °C for the analysis after the cells were seeded in 96-well plates and add polysaccharides for 24 h. Subsequently, the oxidant and antioxidant activity of the cell were examined by MDA, SOD, CAT, GSH and NO with different kits. All these kits were purchased from Nanjing Jiancheng Bioengineering Institute.

The level of ROS was measured with the ROS detection kit, which was purchased from Nanjing Jiancheng Bioengineering Institute, and based on the method for the quantitative detection of extracellular reactive oxygen species with the fluorescent dye of DCFH-DA (2,7dichlorodihydrofluorescein diacetate). The IPEC-J2 cells in 100 μl were seeded in 96-well plates at a density of 1.0 × 10^4^ cells/well. And the 96-well plates were incubated at 37 °C and 5% CO_2_. After 24 h, the EPS and *lacticaseibacillus* were added in the different groups of cells, and incubated for 48 h at 37 °C and 5% CO_2_. The ROS could oxidize non-fluorescent DCFH to produce fluorescent DCF, and its intensity is measured for the level of ROS.

### Real-time quantitative PCR (qRT-PCR) analysis

The qRT-PCR detection System (Bio-Rad) aims to analyze the levels of cytokine gene expression in IPEC-J2. According to the manufacturer’s instructions, the total RNAs were extracted from the cell using a total RNA extraction kit(iNtRON). The extracted RNA was then converted to cDNA using EasyScript First-Strand cDNA Synthesis SuperMix (TransGen Biotech, Beijing, China). The obtained cDNAs were used for real-time PCR with SYBR^®^ qRT-PCR reagent kit (Thermo Scientific, PA, USA). A volume of 1 μL of cDNA was used as the template for each real-time PCR reaction in a total reaction volume of 10 μL. The 10 μL-volume PCR amplification condition was as follows: predenaturation at 95 ℃ for 30 s, then 40 cycles at 95 ℃, for 10 s and 60 ℃ for 30 s.

The Livak method (2-∆∆CT method) was used to calculate the fold change compared to the control group. The fluorescence was determined at 488 nm excitation and 525 nm emission wavelengths. All primer sequences used for qPCR were listed in Table 1.

**Table 1:** Primers used for q-RT PCR for intestinal inflammation and integrity

Primer	Forward sequence	Reverse sequence
β-actin	CCAGTTGGTAACAATGCCATGT	GGCTGTATTCCCCTCCATCG
IL-6	AAAGAGTTGTGCAATGGCAATTCT	AAGTGCATCATCGTTGTTCATACA
IL-10	CGCAGCTCTAGGAGCATGTG	GCTCTTACTGACTGGCATGAG
TNF-ɑ	AGTGGTGCCAGCCGATGGGTTGT	GCTGAGTTGGTCCCCCTTCTCCAG
IFN-γ	AGACAATCAGGCCATCAGCA-	AGACAATCAGGCCATCAGCA-
Occludin	GCTGTGATGTGTGTGAGCTG	GACGGTCTACCTGGAGGAAC
ZO-1	AGGACACCAAAGCATGTGAG	GGCATTCCTGCTGGTTACA
PEDV-M	GGTTCTATTCCCGTTGATGAGGT	AACACAAGAGGCCAAAGTATCCAT

### Indirect immunofluorescence

The cell was fixed with the cell fixation fluid of 4% paraformaldehyde for 300 µL in each well at room temperature (RT) for 10 min, and then the cell was washed for three times with pre-cooled PBS as mentioned before. Then the cells were treated in 3% BSA for 2 h at room temperature. After the permeabilization with 0.4% Triton X-100 at room temperature, the cells were incubated for 45 min at RT with the specific antibody for the anti-PEDV antibody and Anti-F4/80 antibody. And anti-F4/80 antibody is the macrophage marker. The second antibody of goat anti-mouse IgG H&L antibody (FITC) (Abcam) was used in the cell for 30 min at room temperature. Finally, the samples were washed and examined under a fluorescence microscope (Leica, Wetzlar, Germany).

### Experimental analysis of virus titers

The maximum non-toxic dose of EPS was added to a six-well cell culture plate covered with a single layer of IPEC cells, which were incubated at 37 °C for two hours. The PEDV with the virus concentration of 200 PFU/mL was added to the cell culture plate. After incubation for 2 h, the cell was washed for three times with PBS. Then the cell culture plate was added to the agarose of the culture medium of preheating 37 °C, which the liquids are added in equal proportions and a total of 1.5 ml. After the agarose has been sealed in the culture medium, the plates are then placed in a cell incubator for 48 h. The lesion cells were observed under the microscope, and the nutrient agarose containing a neutral red dye (Canspec, Shanghai, China) was added to the culture plate in dark conditions. Then we observed the size, shape, and number of plaques every three hours. Three repeated experiments were set in the course of this experiment, and we also set up a negative control group and a virus control group.

### The effect of copy number of viral RNA

First, IPEC cells were spread in 24-well cell culture plates, and which were allowed to grow about 70–80%. The maximum non-toxic dose of EPS, *lacticaseibacillus* and *lacticaseibacillus* mutant were added in the IPEC-J2, and the PEDV with the virus concentration of 200PFU/mL infected the IPEC-J2 for 48 h. Then we employed real-time qRT-PCR and the CFX96TM real-time PCR detection System (Bio-Rad), aiming to analyze the PEDV mRNA in IPEC-J2. According to the manufacturer's instructions, the total RNAs were extracted from the cell using a total RNA extraction kit(iNtRON). The extracted RNA was then converted to cDNA using EasyScript First-Strand cDNA Synthesis SuperMix (TransGen Biotech, Beijing, China). The obtained cDNAs were used for real-time PCR with SYBR^®^ qRT-PCR reagent kit (Thermo Scientific, PA, USA). TaqMan real-time RT-PCR was used to detect virus numbers. A standard curve was generated by plotting the threshold values against the serially diluted plasmid DNA encoding the PEDV M gene fragment [13]. All primer sequences used for qPCR were listed in Table 1.

### Western blot analysis of IL-10 and STAT3

The antagonist of IL-10 and STAT3 was used in the cell single research of anti-inflammation and the transcription of cytokines, which indicated the mechanism of EPS against the PEDV infection. The IPEC-J2 were lysed with RIPA Lysis and Extraction Buffer (Thermo Fisher, USA) after washing with frozen pre-cooled PBS. Subsequently, the lysis solutions were centrifuged at 12,000 rpm for 5 min at 4 °C, and then the supernatants were transferred into 0.5 mL centrifuge tubes and stored at −20 °C. The protein concentrations of the lysates were measured by BCA protein assay kit (Pierce Chemical Co.). Equal amounts of protein from each cell lysate were separated by loading and electrophoresing on 12% SDS polyacrylamide gel, and the SDS-PAGE was transferred to polyvinylidene fluoride (PVDF) membranes (Millipore, Bio-Rad, USA). The first antibody anti-IL-10 (Solarbio, Beijing, China), anti-pSTAT3 (Solarbio, Beijing, China) were used to target the protein of IL-10, pSTAT3 and Tubulin. The secondary antibody of goat anti-rabbit linked with horseradish peroxidase was used for the detection of the first antibody. After the secondary antibody was applied, the protein was visualized by the ultra-sensitive ECL chemiluminescence kit (Beyotime). The membrane was scanned and the fluorescence density of the bands was analyzed by using NIH Image-J software(National Institutes of Health, Bethesda, MD). Quantified results were presented as the mean ± standard deviation (SD) from three experiments in bar graphs.

## Results

### The antioxidant and differential activities of the EPS

**Fig. 1:**
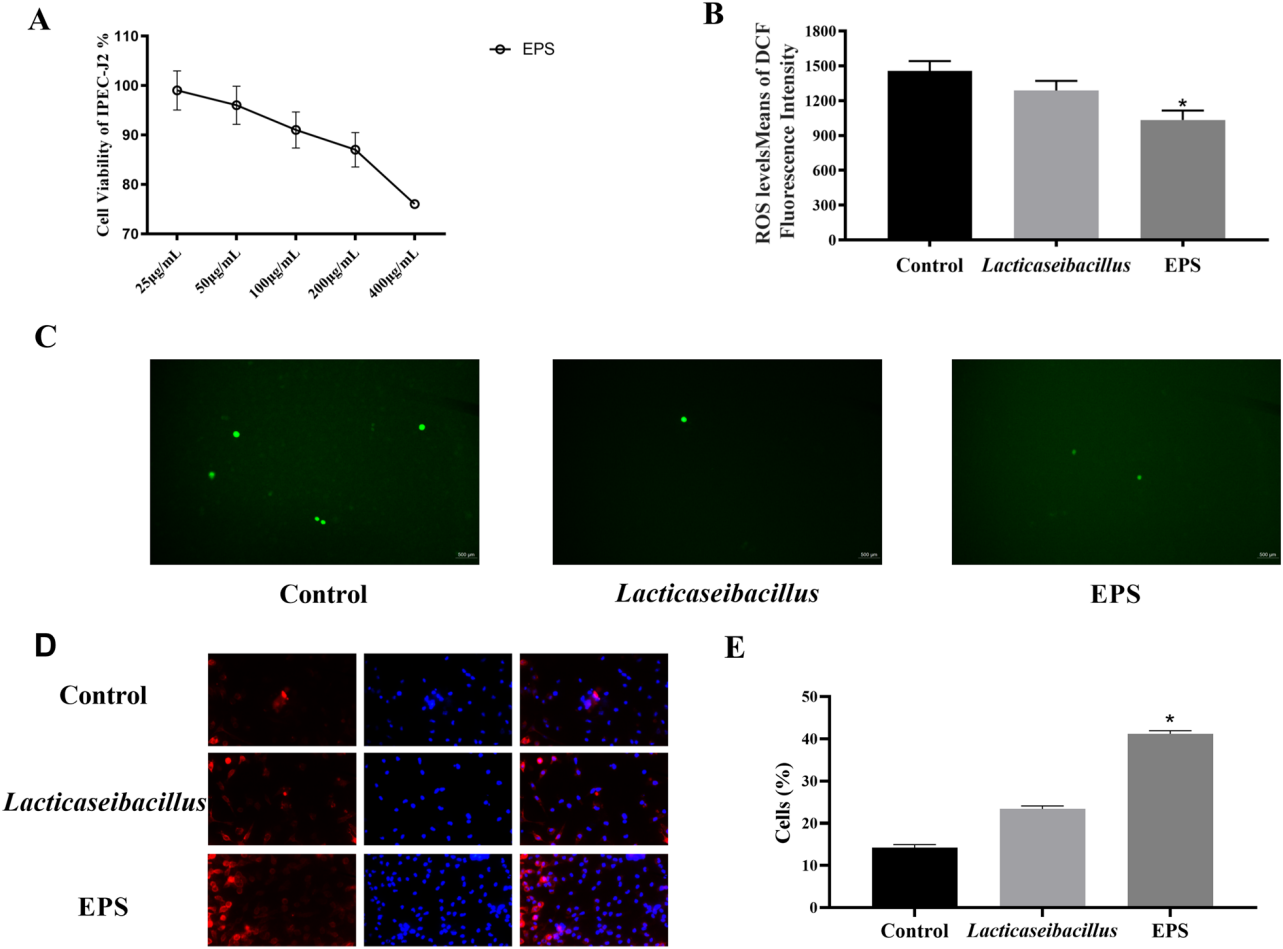
The IPEC-J2 was used to choose the cytotoxicity of EPS from the concentration of 25 mg/mL to 400 mg/mL with the CKK8 method. Comparing with different concentrations, a significant difference was observed in the optimal concentration for our research in the **A**. The ROS was measured with the method for the quantitative detection of extracellular reactive oxygen species with the fluorescent dye of DCFH-DA from the (**B**–**C**). The activities of macrophage were analyzed with the F4/80 antibody and DAPI in the **D**–**E**. The results are represented as the mean ± SEM of three independent experiments. Data with * denote p < 0.05, ** denote p < 0.01

The activity of the maximum dose of cell cytotoxicity test in the IPEC-J2 with the CKK8 method. The experimental data showed that the activity of IPEC cells were decreased with the increasing EPS concentration (Fig. 1A). The results have shown that the cell activity of EPS was gradually decreased in the concentration from 25 μg/mL to 200 μg/mL, and there was no significant difference with cytotoxicity between these concentrations. The maximum cytotoxicity of EPS was 400 μg/mL.

Activities of MDA, CAT, SOD and GSH were used to evaluate oxidant and antioxidant levels for the EPS and *lacticaseibacillus casei* (*lacticaseibacillus*), which are shown in Table 2. Compared with the control group, the results of MDA, CAT and NO were shown that the experimental groups were all lower than the control groups, which indicated that the EPS and *lacticaseibacillus* were with the function of debasing these oxidant lesions in the IPEC. There was no significant difference in the control, EPS and *lacticaseibacillus* groups with the level of MDA. The level of CAT and NO had the same trend in the *lacticaseibacillus* group, which indicated that EPS did not play the role with inhibiting the production of CAT and NO in IPEC. Besides, EPS and *lacticaseibacillus* groups significantly increased the extracellular concentrations of SOD and GSH comparing with the control group. According to these data, we could infer that EPS might be the main functional substance of *lacticaseibacillus* as exerting cellular antioxidant damage.

**Table 2:** Antioxidant Induced by EPS and *Lacticaseibacillus* in IPEC-J2

	MDA (nmol/mg prot)	SOD (U/mg prot)	CAT (U/mg prot)	GSH (μmol/mg prot)	NO (mol/mg prot)
Control	1.21 ± 0.13	2.17 ± 0.23	3.47 ± 0.63	22.62 ± 2.43	3.68 ± 0.38
EPS	1.18 ± 0.14	2.69 ± 0.27*	3.21 ± 0.59	28.72 ± 3.14*	3.32 ± 0.39
*Lacticaseibacillus*	1.17 ± 0.15	2.75 ± 2.22*	2.87 ± 0.61*	27.16 ± 2.34*	3.19 ± 0.42*

The activities of malondialdehyde (MDA), catalase (CAT), superoxide dismutase (SOD) and the level of glutathione (GSH) were used to evaluate antioxidant with IPEC. Activities of SOD and CAT are expressed as units per milligrams of protein (U/mg protein). The activity of GSH and MDA are expressed as μmol/g protein and nmol/mg protein respectively

Data with * denote p < 0.05, ** denote p < 0.01; The results are represented as the mean ± SEM of three independent experiments

The reactive oxygen species are free radicals containing the oxygen atoms, which are formed as natural products of the normal metabolism of oxygen, and have important roles in cell signaling and homeostasis (Fig. 1B, C). Compared with the control group, the extracellular ROS level of the experimental groups, such as EPS group and *lacticaseibacillus* group, could reduce the extracellular ROS level in the IPEC-J2. And the EPS group showed a significant difference comparing with the other two groups. The function of EPS in the immune cell was studied with the immunofluorescence. The activities of macrophage were analyzed with the F4/80 antibody and DAPI in the Fig. 1D–E. The results showed that both of EPS and *lacticaseibacillus* could stimulate the macrophage differentiation, and the effect of EPS was better than the *lacticaseibacillus* with stimulating macrophage differentiation.

**Fig. 2:**
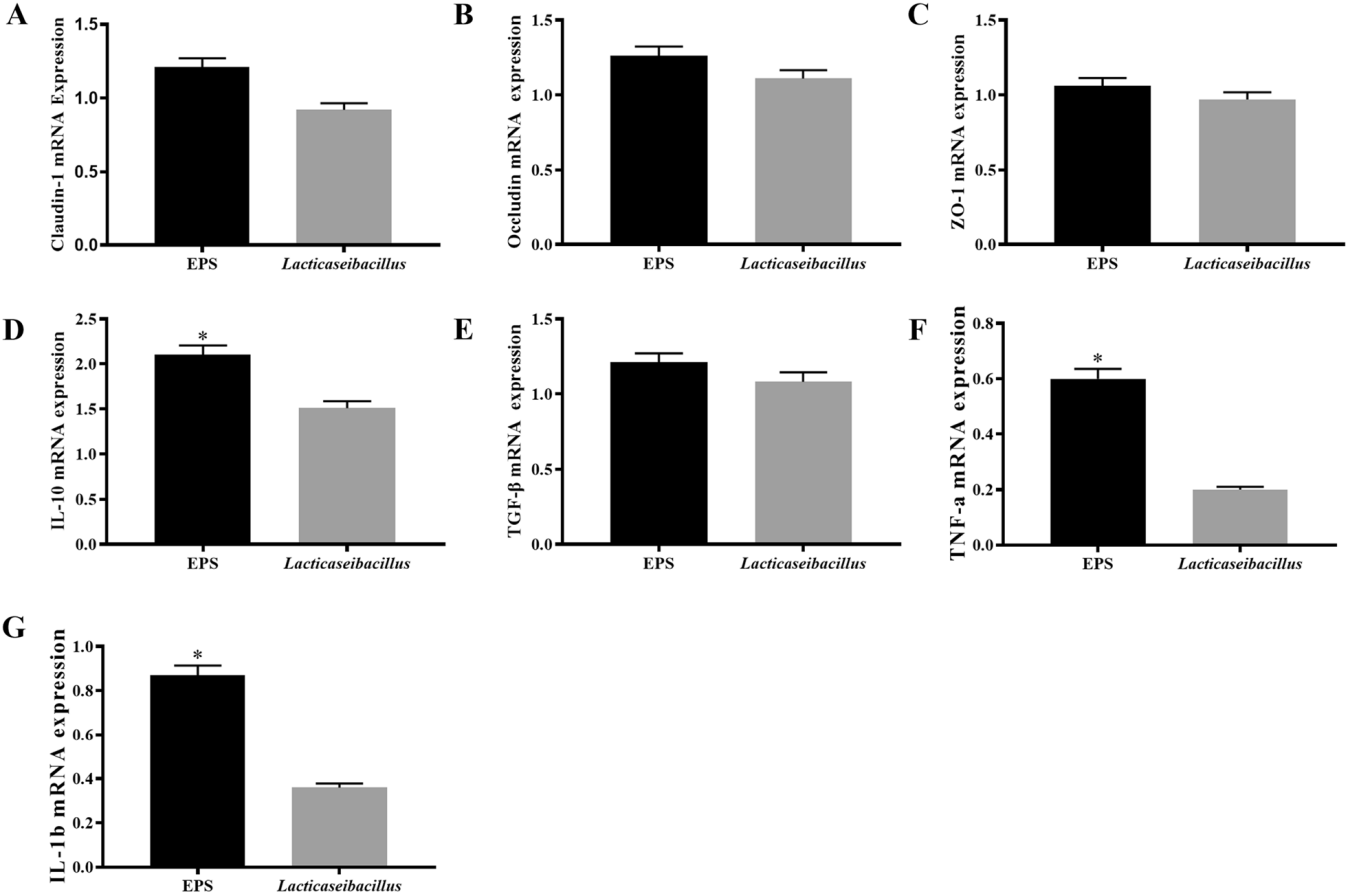
The relative anti-inflammatory and integrated genes were analyzed for the EPS and *Lacticaseibacillus* with the method of qRT-PCR experiments in the level of mRNA expression. The inflammatory and anti-inflammatory genes were IL-1b, TNF-a, IL-10 and TGF-b, and the integrated genes in the IPEC-J2 were Claudin-1, Occludin and ZO-1 with the concentration of 200 mg/mL for EPS and *Lacticaseibacillus* groups. The results are represented as the mean ± SEM of three independent experiments. The Livak method (2-∆∆CT method) was used to calculate the fold change compared to the control group. Data with * denote p < 0.05, ** denote p < 0.01

### Protection of EPS inhibit the inflammation in IPEC

As shown in Fig. 2 the results of RT-PCR indicated that there was no significant difference in the mRNA expression of tight junction protein Claudin-1, Occludin and ZO-1 (Fig. 2A–C) between EPS and *lacticaseibacillus* groups. From the Claudin-1 and Occludin results, the expression of the EPS group was higher than the other groups, but the change was not significant, which indicated that the EPS had a much better effect with maintaining the integrity of epithelial cells than the *lacticaseibacillus* in the IPEC-J2. However, compared with *lacticaseibacillus*, the EPS group had a significant increase in the anti-inflammatory factors both IL-10 and TGF-β (Fig. 2D, E), and IL-10 played the main role in the anti-inflammation comparing with the TGF-β in the EPS groups. However, the EPS also had a promoting effect in the inflammatory cytokines IL-1β (Fig. 2G) and TNF-α (Fig. 2F) comparing with other groups. And the EPS groups were lower than the *lacticaseibacillus* groups, which indicated that the function of lactobacilli against inflammation was mainly exerted through the EPS.

### Genome editing with the EPS gene in *Lacticaseibacillus casei*

**Fig. 3:**
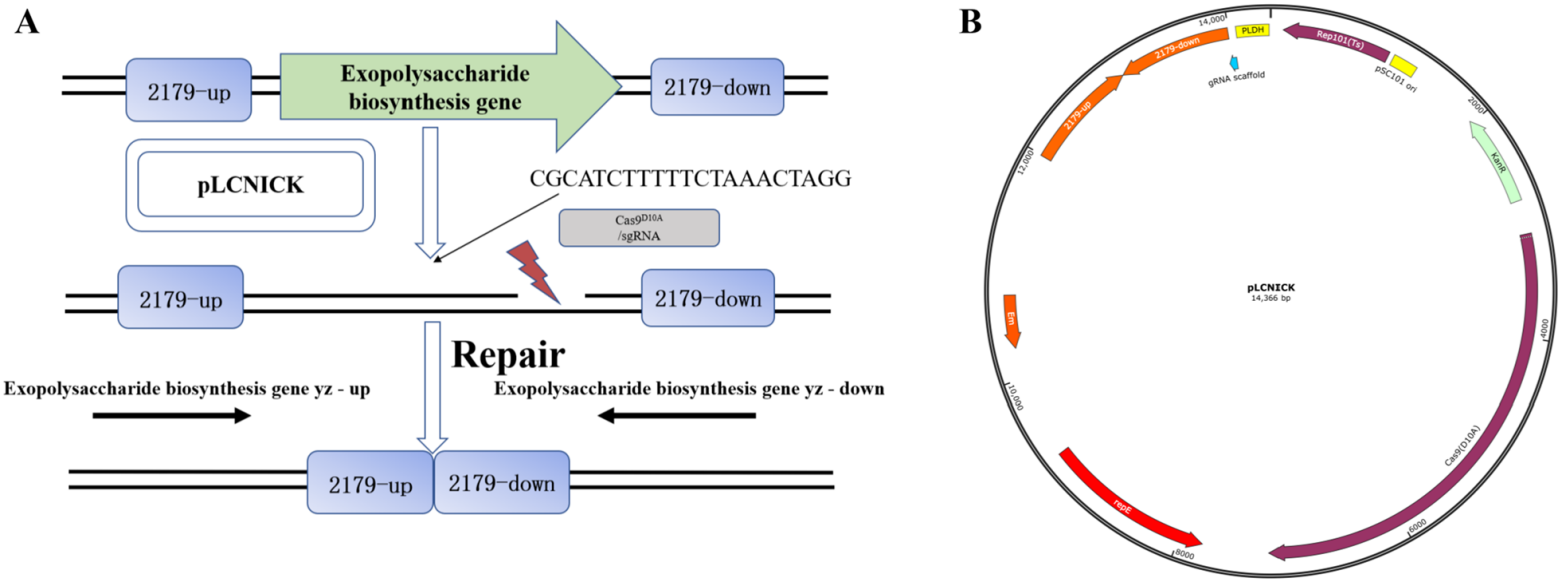
The genome edition for *L. casei* genome engineering based on CRISPR-Cas9^D10A^. A genome editing plasmid deleting the exopolysaccharide biosynthesis gene (glucose-1-phosphate thymidylyltransferase gene, 2179 gene) was constructed in our research in the mechanism diagram (**A**), The positive genome editing plasmid of *L. casei* was constructed, and the **B** shown the diagram losing the glucose-1-phosphate thymidylyltransferase gene, which contain the SgRNA, dCas9, left and right homologous arm genes

An effective, rapid, and precise tool for genome edition, was established for *L. casei* genome engineering based on CRISPR-Cas9. A previous genome editing plasmid deleting the exopolysaccharide biosynthesis gene (glucose-1-phosphate thymidylyltransferase gene, 2179 gene) was studied in our research, which was electrotransformation to obtain positive genome editing *L. casei*. The mechanism of genome engineering *L. casei* based on CRISPR-Cas9 was shown in the Fig. 3A, the pLCNICK plasmid was constructed without the 2179 gene. The sgRNA and Cas9^D10A^ would cut the 2179 gene, and the plasmid repaired and deleted the 2179 gene. The Fig. 3B showed the plasmid of pLCNICK, which contained all the elements in the genome edition of *L. casei*. The positive genome editing *L. casei* was studied the EPS production, and the previous results showed that *L. casei*(2179) mutant decreases the exopolysaccharide (EPS) production about 25% compared with the wild-type *L. casei* when the glucose-1-phosphate thymidylyltransferase gene was deleted. The decreased EPS production of *L. casei*(2179) confirmed the involvement of EPS synthesis, which was used in our next vitro experiment. The genome editing *L. casei* would be much more effective to prove the function of EPS in the vitro.

### The inhibition of PEDV replication with the EPS

**Fig. 4:**
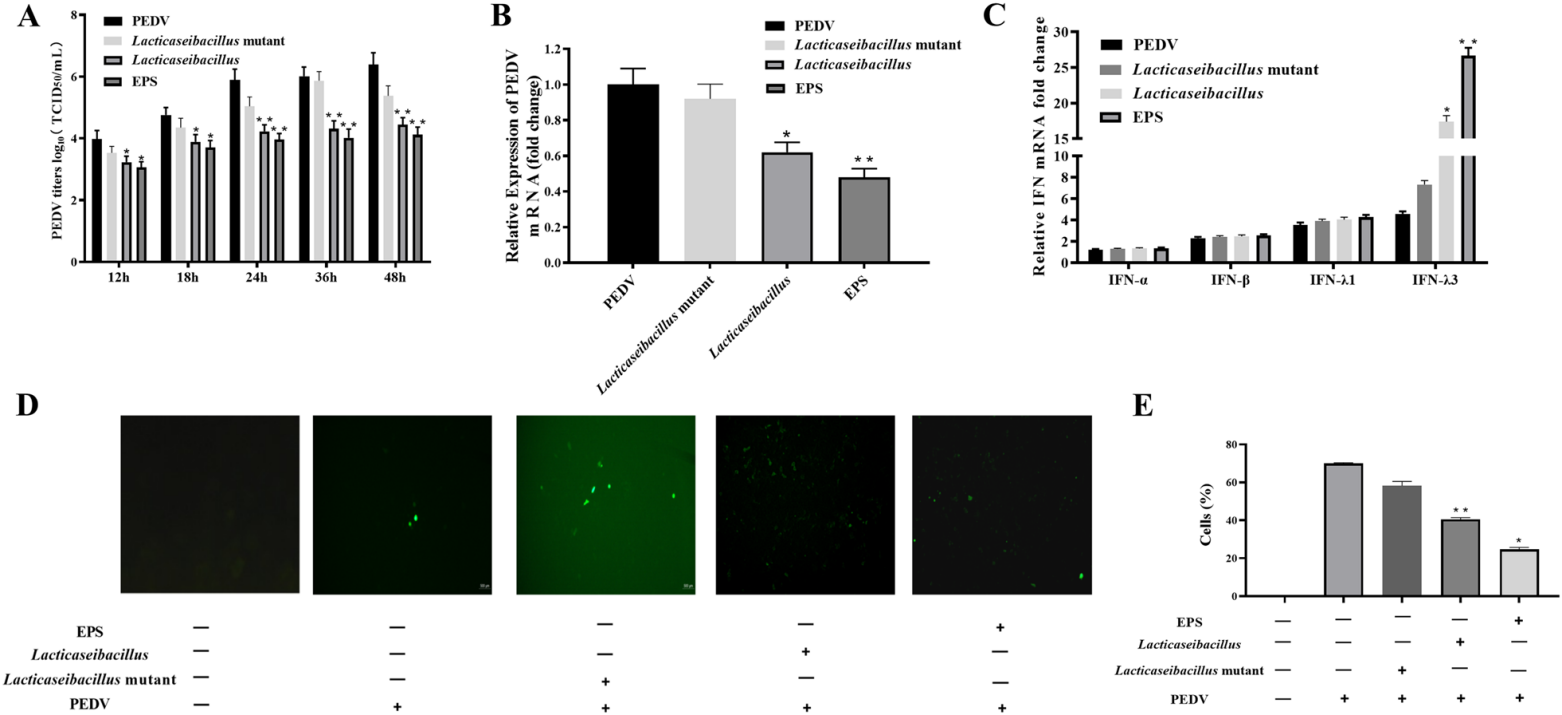
The antiviral effect of EPS, *lacticaseibacillus* and *lacticaseibacillus* mutant was analyzed by plaque assay with TCID50 in the IPEC-J2 in **A**. The TaqMan real-time RT-PCR was used to detect virus mRNA copies numbers in the PEDV replication in **B**. A standard curve was generated by plotting the threshold values against the serially diluted plasmid DNA encoding the PEDV M gene fragment. The C indicated that the type I and type III interferons, such as IFN-α, IFN-β, IFN-λ1 and IFN-λ3, expressed with the mRNA from the IPEC-J2 in the **C**. The indirect immunofluorescence results showed the PEDV distribution patterns detected in the IPEC with the rabbit polyclonal antibodies, which were used to analysis the EPS *lacticaseibacillus* and *lacticaseibacillus* mutant function against PEDV infection in the IPEC-J2 (**D**–**E**). The upper pictures are from the fluorescent (FITC) channel, and the bottom pictures are information added in the cell culture media in each groups. The results are represented as the mean ± SEM of three independent experiments. Data with * denote p < 0.05, ** denote p < 0.01

The antiviral experiment was measured by TCID50 with the maximum non-toxic dose of EPS (200 μg/mL) in Fig. 4A. As shown in the figure, the virus titers were increased in the positive control group and *L. casei*(2179) mutant group with the PEDV infection from 12 to 48 h. The virus titers in the *L. casei*(2179) mutant group was lower than the positive control group with the PEDV infection. However, the EPS, and *lacticaseibacillus* significantly decreased comparing with the PEDV group from the 18 h to 48 h. The experimental results showed that the virus titers and viral RNA copies were significantly decreased with the EPS and *lacticaseibacillus*, and EPS groups had a much better effect against PEDV infection than the *lacticaseibacillus* group. But there was no significance between these two groups. The results indicated that the EPS and *lacticaseibacillus* had the same effect of anti-PEDV infection. And the data showed that EPS was taken the effect against PEDV infection within the *lacticaseibacillus*. The copy number of PEDV mRNA was used to analyze the anti-viral effect of EPS and *lacticaseibacillus* with the qRT-PCR method in Fig. 4B. The experimental results showed that the copy number of PEDV infection was significantly decreased in the group adding *lacticaseibacillus* and EPS. The data showed that the RNA copy number of porcine epidemic diarrhea virus in the EPS and *lacticaseibacillus* group had the same trend comparing with PEDV titers experiments. The EPS could inhibit the RNA and PEDV production.

The EPS and *lacticaseibacillus* were used to analyze the activation promoting the type I and type III interferons, such as IFN-α, IFN-β, IFN-λ1 and IFN-λ3. As shown in the Fig. 4C, there was no significant difference with the gene expression of type I interferon between the EPS, *lacticaseibacillus* and *lacticaseibacillus* mutant groups within the PEDV infection. But the treatment of IPEC-J2 with the EPS, *lacticaseibacillus* and *lacticaseibacillus* mutant have increased in the IFN-α and IFN-β expression comparing with the control group without PEDV infection. The IFN-λ1expression in the EPS group was significantly higher than the other two groups, which means that the EPS could stimulate the IFN-λ1 activation. The most important was that both the EPS and *lacticaseibacillus* could stimulate the IFN-λ3 expression, which expression was the highest comparing with IFN-λ1 against the PEDV infection. The genome editing *L. casei* without EPS lost the specific stimulation with the IFN-λ3 expression, which indicated that the EPS played the role in the *lacticaseibacillus* stimulating the IFN-λs expression. The results of IFN-λ1 and IFN-λ3 indicated that the type III interferon was much more sensitive under the treatment of EPS in the IPEC-J2 against PEDV infection.

We verified the distribution of viral antigens in cells by using a fluorescence method, and divided them into four groups for experiments, such as negative control, PEDV infection, *L. casei* (2179) mutant, *L. casei* and EPS groups with PEDV infection in Fig. 4D–E. Green fluorescence under fluorescent electron microscopy indicated the PEDV in the IPEC-J2 infection. No infection was observed in the negative group, while PEDV infection group and L. casei (2179) mutant were observed in the positive group. Under the observation of fluorescence electron microscope, the fluorescence quantity of EPS groups decreased comparing with the positive group, which shown that EPS and *lacticaseibacillus* inhibited the viral RNA replication and production of the PEDV. The genome editing L. casei without EPS (L. casei(2179) mutant) did not inhibit the PEDV replication and production in the IPEC-J2. The polysaccharides groups, such as EPS and *lacticaseibacillus*, were compared with the amount of green fluorescence, the EPS group was slightly less than the *lacticaseibacillus* group, the effect of anti-PEDV in the EPS group was much stronger than that of the *lacticaseibacillus* group. The pictures are representative of sections derived from each group.

### EPS promote the anti-inflammation and the STAT3 transcription

**Fig. 5:**
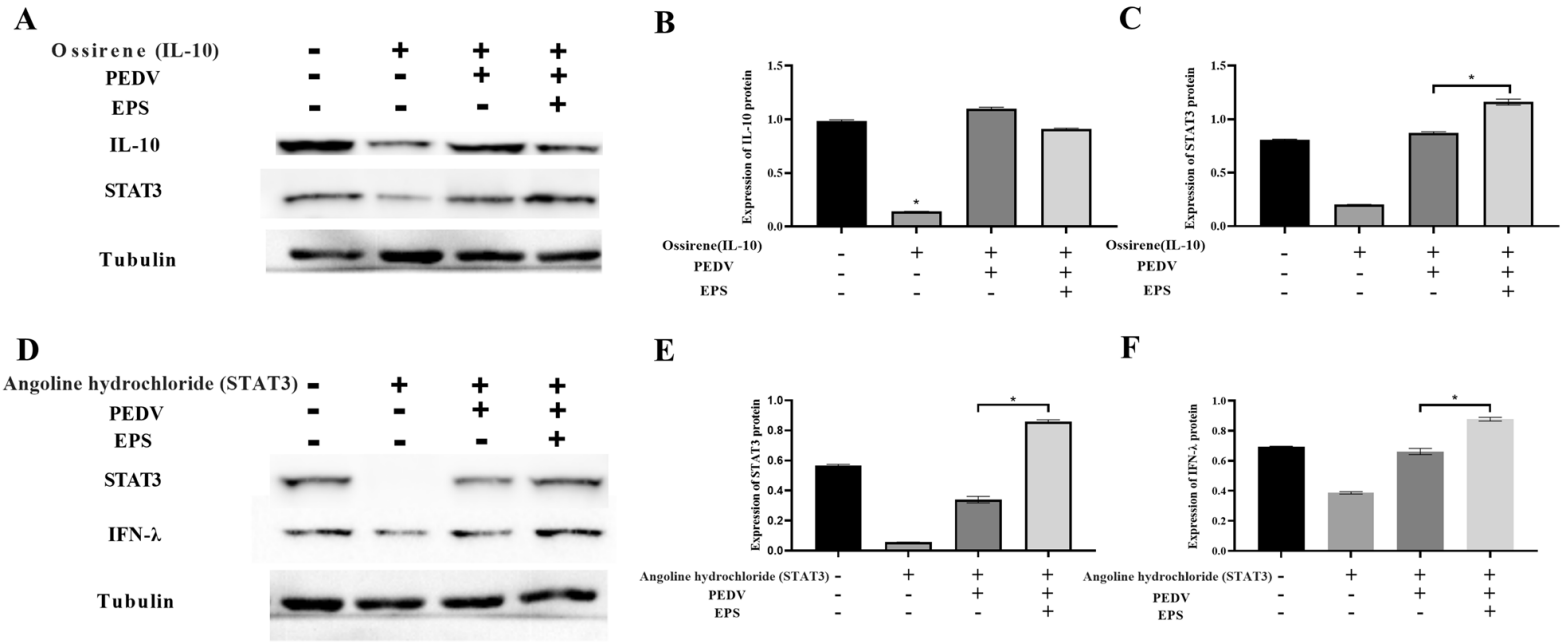
The EPS was the functional component of EPS from *lacticaseibacillus* to the antiviral infection of PEDV with the STAT3 in the cell nucleus, which robust the mRNA transcription of anti-inflammatory and anti-viral genes, such as IL-10 and IFN-λ. The **B** indicated that the EPS did not specifically stimulate the IL-10 single pathways, and enhance the expression STAT3 with the PEDV infection. The **C** explained the higher expression of STAT3 with the EPS stimulation with the PEDV infection comparing with other groups in the IPEC-J2. The EPS from the *lacticaseibacillus* stimulate the STAT3 in the cell nucleus, which specifically robust the mRNA transcription and expression of anti-viral genes with IFN-λ in the **F**

The antagonist of IL-10 and STAT3 were used in the cell single research of anti-inflammation and the transcription of cytokines in Fig. 5. And the antagonist of Ossirene effectively blocked the IL-10 expression in the Fig. 5A. The Western Blot results indicated that there was no significant difference in the expression of anti-inflammatory factors IL-10 between the control group, EPS and PEVD group as shown in Fig. 5B. However, the nuclear transcription factor of STAT3 was also examined in the signal transduction pathways of IL-10 in the Fig. 5A. The relative expression of STAT3 indicated that there was significant difference between the PEDV infectious group and the EPS group, and the relative expression of STAT3 in the EPS group was higher than the PEDV infectious group in the Fig. 5C. The results of IL-10 in the Fig. 5A have indicated that the EPS did not have the function of promoting the IL-10 expression with PEDV infection. However, the relative expression of STAT3 was stimulated by the EPS, which mean that EPS played the role in the nuclear transcription by the STAT3 pathway.

The STAT3 antagonist of Angoline hydrochloride was added in the analysis of the role in the signal transduction pathways of interferon, which was built the connection with type III interferon inducing expression against the PEDV in Fig. 5D. The nuclear transcriptional regulation of STAT3 expression was different from the mechanisms of nuclear transcription with STAT3, which we previously knew in Fig. 5A. The relative expression of STAT3 in the PEDV infecting group was lower than the EPS group with the PEDV infection, which indicated that the EPS could stimulate the STAT3 expression with the PEDV infection under the STAT3 antagonist comparing with the PEDV infecting group in the Fig. 5E. In contrast, under same conditions, the PEVD group conspicuously increased the expression of STAT3 comparing with the STAT3 antagonist group.

In the Fig. 5F, the relative expression of IFN-λ in the EPS group was also higher than the PEDV infectious group, which had the same trend comparing with the mRNA expression of IFN-λ1 and IFN-λ3 in our previous research. The STAT3 and IFN-λ results could include that EPS stimulated STAT3 expression inducing the IFN-λ1 nuclear transcription. According to these experiment results, we could infer that EPS has the effect of anti-inflammation and transcription regulation through the STAT3 single pathway against the PEDV infection.

## Discussion

The EPS has good water soluble with good water-holding capacity, which properties are attributed to the large amounts of hydrogen bond in the structure of polymer chains [29]. Due to the good water holding capacity of EPS, which has been used in our study with various concentrations for the cytotoxic test. According to the present results, the EPS purified from the *lacticaseibacillus* was free from the cytotoxic effects. Our results have shown that the EPS have no cytotoxic effects with different concentrations from 25 μg to 200 μg/mL on the IPEC-J2. However, there was significant difference between the concentration 200 μg/mL to 400 μg/mL in the cellular viability inhibition percentage and cellular proliferation comparing with the non-treated cells. These results were similar to the further research of purifying EPS from *lacticaseibacillus* acidophilus 20079[8]. Besides, the EPS are biodegradable, non-toxic, bio-compatible, and antioxidant activities in a natural source. Both of the EPS and *lacticaseibacillus* in our study were with the properties of antioxidant activities, and the EPS was much better than the *lacticaseibacillus* in the clear the CAT and NO, which enhanced the SOD and GSH. EPS purified from different strain *lacticaseibacillus*, which had different derivatives of EPS from *lacticaseibacillus* with different antioxidant property, such as *lacticaseibacillus delbrueckii* and *bulgaricus*, which was mainly based on the structural characterization [43]. Most interestingly, the antioxidant property of EPS took a role in the various cell lines of different hosts. Researchers have taken a great number of studies on the exopolysaccharides following the antioxidant property in different diseases, such as inflammatory bowel disease symptoms, antibacterial activities, colon cancer and Alzheimer's disease [2, 35, 38]. However, there was no research to focus on the character of innate immunity of antiviral infection. The macrophage differentiation results indicated that EPS had taken the function of stimulating the immune cell differentiation. Previous studies has found that UV-killed *lacticaseibacillus* strains and their EPSs trigger a Th1-type immune response in a human host [23], and UV-killed *lacticaseibacillus* strains through TLR4 and TLR2 signal grouping mediated immunological properties [24]. Certainly, components mediated the immunomodulation, and evaluated the signaling mechanism, which contain the LPS, surface layer protein, SLP; genomic DNA, gDNA, CpG-ODN [36]. The clear signaling mechanism and application were not clear in the immune regulation.

The epithelial cell infection, such as coronaviruses, SARS-CoV and human respiratory syncytial (HRSV), produced a high level of ROS, associated with cytokine production, inflammation and cell death pathological processes. Investigation of virus infections on ROS-producing indicated that ROS are crucial for replication of virus-associated disease [22]. The previous studies have supported that there was a tight connection between the concentration and species of *lacticaseibacillus* inhibiting the production of ROS in response to challenge with lactic bacteria [25]. ROS entities are extremely toxic, and have deleterious, such as fungi, parasites, and bacteria. Investigation of the influence of these infections on ROS-producing and ROS-scavenging enzymes, and systems may allow for the identification of those that are crucial for replication of the pathogens and occurrence of virus-associated disease. And the ingredients of heat-killed *lacticaseibacillus* would also play the role of effective constituent on the bacterium composition. The *lacticaseibacillus* was with the function of relieving the ROS in various infections. The immunological role of the EPS decreasing ROS production has been highlighted in defecting effects in the IPEC-J2, and the EPS has a better effect than *lacticaseibacillus* in inhibiting the production of ROS. Likewise, the inhibition of new ROS molecules in response to *lacticaseibacillus* was associated with the mechanisms underlying anti-inflammation and anti-virus.

There was no relative research on the anti-inflammation and maintenance of the integrity of epithelia in the intestinal epithelial cell. Our results have shown that the EPS take a much better effect in reducing the expression of inflammatory cytokines, such as IL-1β and TNF-α in the IPEC-J2. And the EPS has much more important role to stimulate the expression of the anti-inflammatory cytokines, such as TGF-β and IL-10, which was significantly higher than the *lacticaseibacillus* [32]. All of these results indicated that the EPS was mainly stimulating the expression of the anti-inflammatory cytokines, but not the expression of the inflammatory cytokines. Most importantly, the EPS could stimulate the type III IFN-λ expression, which was with the specificity expression in the IPEC-J2. *L. salivarius* isolating from the intestine of wakame-fed pigs maintained their ability to enhance IFN-β and IFN-λ, which played the anti-viral factors expression in porcine intestinal epithelial cells [15]. The freeze-dried compound of *lacticaseibacillus kefiri* stimulated the production of antiviral cytokines, such as IFN-α and IFN-λ in DCs, which active ingredients was not clear with a unique composition and functionality in the expression of cytokines [11]. Our research firstly found the transcriptional mechanism of the IFN single pathway in the intestinal epithelial cells, which was deeply studied to build the connection between EPS and anti-virus activity. Many anti-viral and anti-inflammatory researches were connected with the *lacticaseibacillus* through the innate immunity, but these studies were not clear for the active ingredients and cell singing. The most important was that there was rarely research on the cell EPS from the *lacticaseibacillus* against animal coronavirus infection. The probiotics was effectively used to rebalance the intestinal microbiome for controlling COVID-19, which obtained with immunomodulatory ability, and mimicked the blood cytokine environment produced in early immune responses of COVID-19 infection [20].

Previous study found that the PEDV could activate the transcription of type I interferon in porcine monocyte-derived dendritic cells, and the PEDV did not replicate in the dendritic cells [37, 46]. However, the E protein in the PEDV acts as an antagonist suppressing IFN-β through the RIG-I-mediated signaling in the host innate immune response of IPEC-J2 [50]. There were much more researchers found that the PEDV escape the type I interferon in the various singles molecule [5]. Recently, type III interferon was considered that it could inhibit the PEDV infection in the IPEC-J2, which have a much better effect than the IFN-α inhibiting the PEDV replication under the ISGs and STING in the host cell [49], and there was not complete overlap between the unique transcriptional profile inducing by the IFN-λ3 and IFN-α [30]. It was regarding that the elicited nuclear transcription factor were the difference between the type I and III IFNs in restricting porcine intestinal viral infection with the RNA replication of PEDV. The *Lacticaseibacillus plantarum* metabolites was experimented to determine the inhibitory effects on PEDV replication and prevented PEDV adsorption, and also alleviated inflammatory response. The metabolites of EPS was one of the main components from the *lacticaseibacillus* [14]. Moreover, the extracellular extracts or cell wall fractions of *lactic acid bacteria* could enhance the found that cell viabilities decreased mRNA expression of PEDV in the Vero cells, and which decreased the mRNA expression of TNF-α and IL-8. And the extracellular extracts of *Ln. mesenteroides* YPK30 possessed in vitro prophylactic, therapeutic, and direct-inhibitory effects against PEDV in the Vero cell model [4]. The extracts or cell wall fractions of YM22 and YM33 strains for 24 h before infection with PEDV showed significantly higher cell viabilities and lower mRNA expression of PEDV nucleocapsid (PEDV-N) than the unpretreated cells, indicating that the extracellular extracts and cell wall fractions of YM22 and YM33 possessed prophylactic effects on Vero cells against PEDV infection.

The previous studies have shown that the treatment with *lacticaseibacillus* caused an increase in the specific and total serum IgA titer on dendritic cells (DCs), was also stimulated the production of antiviral cytokines, IFN-α and IFN-λ(IL29) in DCs [11]. But there were not much more researches on the mechanism on how dose the *lacticaseibacillus* stimulated the DC expressing the IFN-λ, and enhanced the IFN-λ expressing in the epithelial cell. The polysaccharide from Ginkgo biloba exocarp possessed an effective inhibitory effect on viral attachment and entry steps of the PEDV life cycle [28]. Our results firstly explained the anti-viral and anti-inflammatory mechanism with the EPS produced *lacticaseibacillus* against the PEDV infection in the IPEC-J2. The EPS could enhance the IL-10 expression in the PEDV infection, and increased the STAT3 expression in the PEDV infection. The blocker of STAT3 in the experiments shown that the EPS was with the dual function, increasing the IL-10 and the STAT3 expression, against the inflammatory storm with the PEDV infection. The most important was that the previous study defined STAT3 as a negative regulator of type I IFN response, and provided a therapeutic target for viral infections [45]. But our study found that the EPS could specifically stimulate IFN-λ expression with the STAT3 single pathway against the PEDV infection. In the classical Jak-Stat antiviral signaling pathway, the inflammatory STAT3-HNF4α feedback loop activating IFN-λ1 inhibits HCV replication through the suppression of miRNA-122 [1]. Besides, the significant anti-viral activity of IL-22 could be a novel therapeutic against multiple enteric diarrhea viruses in IPEC-J2, such as PEDV, PoRV and TGEV, which were mediated by the activation of the STAT3 signal pathway [48]. The secreted factors and cell surface components from *lacticaseibacillus* species have been shown to modulate the host immune system. However, the precise role of *L. reuteri* surface proteins in influencing dendritic cells (DCs) could promote IL-10 and suppress inflammation [9, 42]. The IL-10 receptor complex is the first step for the EPS in initiating IL-10 signaling pathways that regulates intestinal inflammation and viral persistence. However, these results did not explain the IFN-λ overexpression, and the role of STAT3 comparing with IL-10 in the PEDV infection. After the infection by the SARS-CoV-2 proteins, NSP1, and ORF6, STAT1. With STAT1 activity restricted, STAT3 then becomes dominant and induces STAT3- ISGs. Both STAT1 and STAT3 activity is inhibited, which was also inhibited the kinase activity of JAKs for the negative feedback of IFN-I signaling [33].

The previous studies hold that type I IFNs increased the anti-inflammatory IL-10 and decreased pro-inflammatory cytokines in the virus infection, which could also explain that the type I IFNs was not effective against the virus replication with the PEDV infection. The timing of IL-10 expression and production with the EPS in IPEC-J2 during the PEDV infection could be particularly important for the IFN-λ expression in the future. Type I IFNs acted via the IFNα/β receptor (IFNAR)1 and IFNAR2, but the type III IFN signals was active through IFNLR1 and IL-10R2, all the phenomenon and results implied that the type III IFN signals induced formation of a receptor complexed between IL10R2 and IL-10. The most interestingly, the STAT3 primarily homodimerize built the connection between IL-10R2 and type III IFN. and the increasing the expression of IL-10R1, and subsequently IL-10 induced a higher level of STAT3 on monocytes. The IL-29, a member of the type III IFN family, was sensitized to IL-10 stimulation in APCs [31]. If the virus has already compromised STAT1, then treatment with the EPS producing from *lacticaseibacillus* focus on preventing the excessive activation of STAT3 that drives the remission of proinflammatory cytokines and increasing IFN-λ1 to inhibit the PEDV infection.

## Conclusion

This study firstly focused on the EPS-producing *lacticaseibacillus* with the gene editing *lacticaseibacillus* and purification, both of which have displayed the potential antioxidation and anti-inflammation. The immunomodulatory activities were highlight the immunomodulatory potential of EPS against the PEDV infection. All these results indicated the detailed mechanism of activating type III IFN signals receptor of IL-10R2 inhibiting the replication and inflammatory storm within coronavirus PEDV.

## Data Availability

The raw data supporting the conclusions of this article will be made available by the corresponding authors, without undue reservation.

## References

[1] Aboulnasr F, Hazari S, Nayak S, Chandra PK, Panigrahi R, Ferraris P, Chava S, Kurt R, Song K, Dash A, Balart LA, Garry RF, Wu T, Dash S (2015). IFN-lambda inhibits MiR-122 transcription through a Stat3-HNF4alpha inflammatory feedback loop in an IFN-alpha resistant HCV cell culture system. PLoS ONE.

[2] Adebayo-Tayo B, Fashogbon R (2020). In vitro antioxidant, antibacterial, in vivo immunomodulatory, antitumor and hematological potential of exopolysaccharide produced by wild type and mutant *Lactobacillus*
*delbureckii* subsp. bulgaricus. Heliyon.

[3] Balzaretti S, Taverniti V, Guglielmetti S, Fiore W, Minuzzo M, Ngo HN, Ngere JB, Sadiq S, Humphreys PN, Laws AP (2017). A novel rhamnose-rich hetero-exopolysaccharide isolated from *Lactobacillus*
*paracasei* DG activates THP-1 human monocytic cells. Appl Environ Microbiol.

[4] Chang-Liao WP, Lee A, Chiu YH, Chang HW, Liu JR (2020). Isolation of a *Leuconostoc*
*mesenteroides* strain with anti-porcine epidemic diarrhea virus activities from kefir grains. Front Microbiol.

[5] Chang CY, Liu HM, Chang MF, Chang SC (2020). Middle east respiratory syndrome coronavirus nucleocapsid protein suppresses type I and type III interferon induction by targeting RIG-I signaling. J Virol.

[6] Chen S, He X, Qin Z, Li G, Wang W, Nai Z, Tian Y, Liu D, Jiang X (2023). Loss in the antibacterial ability of a pyrr gene regulating pyrimidine biosynthesis after using crispr/cas9-mediated knockout for metabolic engineering in lactobacillus casei. Microorganisms..

[7] Chen YM, Limaye A, Chang HW, Liu JR (2022). Screening of lactic acid bacterial strains with antiviral activity against porcine epidemic diarrhea. Probiotics Antimicrob Proteins.

[8] El-Deeb NM, Yassin AM, Al-Madboly LA, El-Hawiet A (2018). A novel purified *Lactobacillus*
*acidophilus* 20079 exopolysaccharide, LA-EPS-20079, molecularly regulates both apoptotic and NF-kappaB inflammatory pathways in human colon cancer. Microb Cell Fact.

[9] Engevik MA, Ruan W, Esparza M, Fultz R, Shi Z, Engevik KA, Engevik AC, Ihekweazu FD, Visuthranukul C, Venable S, Schady DA, Versalovic J (2021). Immunomodulation of dendritic cells by *Lactobacillus*
*reuteri* surface components and metabolites. Physiol Rep.

[10] Felgenhauer U, Schoen A, Gad HH, Hartmann R, Schaubmar AR, Failing K, Drosten C, Weber F (2020). Inhibition of SARS-CoV-2 by type I and type III interferons. J Biol Chem.

[11] Ghoneum M, Felo N, Agrawal S, Agrawal A (2015). A novel kefir product (PFT) activates dendritic cells to induce CD4+T and CD8+T cell responses in vitro. Int J Immunopathol Pharmacol.

[12] Gorska S, Hermanova P, Ciekot J, Schwarzer M, Srutkova D, Brzozowska E, Kozakova H, Gamian A (2016). Chemical characterization and immunomodulatory properties of polysaccharides isolated from probiotic *Lactobacillus*
*casei* LOCK 0919. Glycobiology.

[13] Hou X, Jiang X, Jiang Y, Tang L, Xu Y, Qiao X, Min L, Wen C, Ma G, Li Y (2018). Oral immunization against PEDV with recombinant *Lactobacillus*
*casei* expressing dendritic cell-targeting peptide fusing COE protein of PEDV in piglets. Viruses.

[14] Huang S, Yu Q, Xie L, Ran L, Wang K, Yang Y, Gan L, Song Z (2021). Inhibitory effects of *Lactobacillus*
*plantarum* metabolites on porcine epidemic diarrhea virus replication. Res Vet Sci.

[15] Indo Y, Kitahara S, Tomokiyo M, Araki S, Islam MA, Zhou B, Albarracin L, Miyazaki A, Ikeda-Ohtsubo W, Nochi T, Takenouchi T, Uenishi H, Aso H, Takahashi H, Kurata S, Villena J, Kitazawa H (2021). *Ligilactobacillus*
*salivarius* strains isolated from the porcine gut modulate innate immune responses in epithelial cells and improve protection against intestinal viral-bacterial superinfection. Front Immunol.

[16] Jafarzadeh A, Nemati M, Saha B, Bansode YD, Jafarzadeh S (2020). Protective potentials of type III interferons in COVID-19 patients: lessons from differential properties of type I- and III interferons. Viral Immunol.

[17] Jiang X, Gu S, Liu D, Zhao L, Xia S, He X, Chen H, Ge J (2018). Lactobacillus brevis 23017 relieves mercury toxicity in the colon by modulation of oxidative stress and inflammation through the interplay of MAPK and NF-kappaB signaling cascades. Front Microbiol.

[18] Jiang X, Hou X, Tang L, Jiang Y, Ma G, Li Y (2016). A phase trial of the oral Lactobacillus casei vaccine polarizes Th2 cell immunity against transmissible gastroenteritis coronavirus infection. Appl Microbiol Biotechnol.

[19] Jiang X, Yu M, Qiao X, Liu M, Tang L, Jiang Y, Cui W, Li Y (2014). Up-regulation of MDP and tuftsin gene expression in Th1 and Th17 cells as an adjuvant for an oral Lactobacillus casei vaccine against anti-transmissible gastroenteritis virus. Appl Microbiol Biotechnol.

[20] Kageyama Y, Nishizaki Y, Aida K, Yayama K, Ebisui T, Akiyama T, Nakamura T (2022). *Lactobacillus*
*plantarum* induces innate cytokine responses that potentially provide a protective benefit against COVID-19: a single-arm, double-blind, prospective trial combined with an in vitro cytokine response assay. Exp Ther Med.

[21] Kaji R, Kiyoshima-Shibata J, Nagaoka M, Nanno M, Shida K (2010). Bacterial teichoic acids reverse predominant IL-12 production induced by certain lactobacillus strains into predominant IL-10 production via TLR2-dependent ERK activation in macrophages. J Immunol.

[22] Khomich OA, Kochetkov SN, Bartosch B, Ivanov AV (2018). Redox biology of respiratory viral infections. Viruses.

[23] Kishimoto M, Nomoto R, Mizuno M, Osawa R (2017). An in vitro investigation of immunomodulatory properties of *Lactobacillus*
*plantarum* and L. delbrueckii cells and their extracellular polysaccharides. Biosci Microbiota Food Health.

[24] Kishimoto M, Nomoto R, Osawa R (2015). In vitro evaluation of immunological properties of extracellular polysaccharides produced by Lactobacillus delbrueckii strains. Biosci Microbiota Food Health.

[25] Kong Y, Olejar KJ, On SLW, Chelikani V (2020). The potential of Lactobacillus spp. for modulating oxidative stress in the gastrointestinal tract. Antioxidants (Basel).

[26] Lai HH, Chiu CH, Kong MS, Chang CJ, Chen CC (2019). Probiotic *Lactobacillus*
*casei*: effective for managing childhood diarrhea by altering gut microbiota and attenuating fecal inflammatory markers. Nutrients.

[27] Lai W, Wang C, Yu F, Lu L, Wang Q, Jiang X, Xu X, Zhang T, Wu S, Zheng X, Zhang Z, Dong F, Jiang S, Liu K (2016). An effective strategy for recapitulating N-terminal heptad repeat trimers in enveloped virus surface glycoproteins for therapeutic applications. Chem Sci.

[28] Lee JH, Park JS, Lee SW, Hwang SY, Young BE, Choi HJ (2015). Porcine epidemic diarrhea virus infection: inhibition by polysaccharide from Ginkgo biloba exocarp and mode of its action. Virus Res.

[29] Lee W, Lee SH, Ahn DG, Cho H, Sung MH, Han SH, Oh JW (2013). The antiviral activity of poly-gamma-glutamic acid, a polypeptide secreted by Bacillus sp., through induction of CD14-dependent type I interferon responses. Biomaterials.

[30] Li L, Xue M, Fu F, Yin L, Feng L, Liu P (2019). IFN-Lambda 3 mediates antiviral protection against porcine epidemic diarrhea virus by inducing a distinct antiviral transcript profile in porcine intestinal epithelia. Front Immunol.

[31] Liu BS, Janssen HL, Boonstra A (2012). Type I and III interferons enhance IL-10R expression on human monocytes and macrophages, resulting in IL-10-mediated suppression of TLR-induced IL-12. Eur J Immunol.

[32] Liu C, Choi MW, Xue X, Cheung PCK (2019). Immunomodulatory effect of structurally characterized mushroom sclerotial polysaccharides isolated from Polyporus rhinocerus on bone marrow dendritic cells. J Agric Food Chem.

[33] Matsuyama T, Kubli SP, Yoshinaga SK, Pfeffer K, Mak TW (2020). An aberrant STAT pathway is central to COVID-19. Cell Death Differ.

[34] Mendez Y, Chang J, Humpierre AR, Zanuy A, Garrido R, Vasco AV, Pedroso J, Santana D, Rodriguez LM, Garcia-Rivera D, Valdes Y, Verez-Bencomo V, Rivera DG (2018). Multicomponent polysaccharide-protein bioconjugation in the development of antibacterial glycoconjugate vaccine candidates. Chem Sci.

[35] Min Z, Xiaona H, Aziz T, Jian Z, Zhennai Y (2020). Exopolysaccharides from Lactobacillus plantarum YW11 improve immune response and ameliorate inflammatory bowel disease symptoms. Acta Biochim Pol.

[36] Qi SR, Cui YJ, Liu JX, Luo X, Wang HF (2020). Lactobacillus rhamnosus GG components, SLP, gDNA and CpG, exert protective effects on mouse macrophages upon lipopolysaccharide challenge. Lett Appl Microbiol.

[37] Qin Z, Nai Z, Li G, He X, Wang W, Xia J, Chao W, Li L, Jiang X, Liu D (2023). The Oral Inactivated Porcine Epidemic Diarrhea Virus Presenting in the Intestine Induces Mucosal Immunity in Mice with Alginate-Chitosan Microcapsules. Animals.

[38] Sirin S, Aslim B (2020). Characterization of lactic acid bacteria derived exopolysaccharides for use as a defined neuroprotective agent against amyloid beta1-42-induced apoptosis in SH-SY5Y cells. Sci Rep.

[39] Smits HH, Engering A, van der Kleij D, de Jong EC, Schipper K, van Capel TM, Zaat BA, Yazdanbakhsh M, Wierenga EA, van Kooyk Y, Kapsenberg ML (2005). Selective probiotic bacteria induce IL-10-producing regulatory T cells in vitro by modulating dendritic cell function through dendritic cell-specific intercellular adhesion molecule 3-grabbing nonintegrin. J Allergy Clin Immunol.

[40] Song X, Huang H, Xiong Z, Ai L, Yang S (2017). CRISPR-Cas 9(D10A) nickase-assisted genome editing in* Lactobacillus** casei*. Appl Environ Microbiol.

[41] Sung HW, Chen CN, Chang Y, Liang HF (2002). Biocompatibility study of biological tissues fixed by a natural compound (reuterin) produced by *Lactobacillus*
*reuteri*. Biomaterials.

[42] Sung HW, Chen CN, Liang HF, Hong MH (2003). A natural compound (reuterin) produced by *Lactobacillus*
*reuteri* for biological-tissue fixation. Biomaterials.

[43] Tang W, Dong M, Wang W, Han S, Rui X, Chen X, Jiang M, Zhang Q, Wu J, Li W (2017). Structural characterization and antioxidant property of released exopolysaccharides from *Lactobacillus*
*delbrueckii* ssp. bulgaricus SRFM-1. Carbohydr Polym.

[44] Wang C, Shan L, Qu S, Xue M, Wang K, Fu F, Wang L, Wang Z, Feng L, Xu W, Liu P (2020). The coronavirus PEDV evades type III interferon response through the miR-30c-5p/SOCS1 Axis. Front Microbiol.

[45] Wang WB, Levy DE, Lee CK (2011). STAT3 negatively regulates type I IFN-mediated antiviral response. J Immunol.

[46] Wang X, Ohnstad M, Nelsen A, Nelson E (2017). Porcine epidemic diarrhea virus does not replicate in porcine monocyte-derived dendritic cells, but activates the transcription of type I interferon and chemokine. Vet Microbiol.

[47] Xu K, Cai H, Shen Y, Ni Q, Chen Y, Hu S, Li J, Wang H, Yu L, Huang H, Qiu Y, Wei G, Fang Q, Zhou J, Sheng J, Liang T, Li L (2020). Management of corona virus disease-19 (COVID-19): the Zhejiang experience. Zhejiang Da Xue Xue Bao Yi Xue Ban.

[48] Xue M, Zhao J, Ying L, Fu F, Li L, Ma Y, Shi H, Zhang J, Feng L, Liu P (2017). IL-22 suppresses the infection of porcine enteric coronaviruses and rotavirus by activating STAT3 signal pathway. Antiviral Res.

[49] Zhao M, Li L, Zhai L, Yue Q, Liu H, Ren S, Jiang X, Gao F, Bai S, Li H, Zhang Y, Xu H, Zhang L, Liu P, Tan M, Yu Q (2020). Comparative transcriptomic and proteomic analyses prove that IFN-lambda1 is a more potent inducer of ISGs than IFN-alpha against porcine epidemic diarrhea virus in porcine intestinal epithelial cells. J Proteome Res.

[50] Zheng L, Wang X, Guo D, Cao J, Cheng L, Li X, Zou D, Zhang Y, Xu J, Wu X, Shen Y, Wang H, Yu W, Li L, Xiao L, Song B, Ma J, Liu X, Li P, Xu S, Xu X, Zhang H, Wu Z, Cao H (2021). Porcine epidemic diarrhea virus E protein suppresses RIG-I signaling-mediated interferon-beta production. Vet Microbiol.

[51] Zhou P, Fan H, Lan T, Yang XL, Shi WF, Zhang W, Zhu Y, Zhang YW, Xie QM, Mani S, Zheng XS, Li B, Li JM, Guo H, Pei GQ, An XP, Chen JW, Zhou L, Mai KJ, Wu ZX, Li D, Anderson DE, Zhang LB, Li SY, Mi ZQ, He TT, Cong F, Guo PJ, Huang R, Luo Y, Liu XL, Chen J, Huang Y, Sun Q, Zhang XL, Wang YY, Xing SZ, Chen YS, Sun Y, Li J, Daszak P, Wang LF, Shi ZL, Tong YG, Ma JY (2018). Fatal swine acute diarrhoea syndrome caused by an HKU2-related coronavirus of bat origin. Nature.

